# Disordered Eating Behaviors Are Associated with Gestational Weight Gain in Adolescents

**DOI:** 10.3390/nu13093186

**Published:** 2021-09-13

**Authors:** Reyna Sámano, Luis Ortiz-Hernández, Hugo Martínez-Rojano, Oralia Nájera-Medina, Gabriela Chico-Barba, Bernarda Sánchez-Jiménez, Jessica Cruz-Cruz, María José Echenique-González

**Affiliations:** 1 Programa de Posgrado Doctorado en Ciencias Biológicas y de la Salud, División de Ciencias Biológicas y de la Salud, Universidad Autónoma Metropolitana, Mexico City 04960, Mexico; onajera@correo.xoc.uam.mx; 2 Departamento de Nutrición y Bioprogramación, Instituto Nacional de Perinatología, Secretaría de Salud, Mexico City 11000, Mexico; gabyc3@gmail.com (G.C.-B.); emiberna20@yahoo.com.mx (B.S.-J.); 3 Departamento de Atención a la Salud, Universidad Autónoma Metropolitana Xochimilco, Mexico City 04960, Mexico; 4 Sección de Posgrado e Investigación de la Escuela Superior de Medicina del Instituto Politécnico Nacional, Mexico City 11340, Mexico; hmartinez_59@yahoo.com.mx; 5 Escuela de Ciencias de la Salud, Universidad del Valle de México-Chapultepec, Mexico City 11810, Mexico; jessy.pink99@gmail.com; 6 Departamento de Salud, Nutrición y Ciencias de los Alimentos, Universidad Iberoamericana-Ciudad de México, Mexico City 01219, Mexico; mariajo_126@hotmail.com

**Keywords:** teenage pregnancy, risky feeding behavior, child nutrition, gestational weight gain, disordered eating behavior, Mexico

## Abstract

Disordered eating behaviors (DEBs) and adolescent pregnancy are public health problems. Among adolescents, there is little evidence concerning the relationship of DEB with gestational weight gain (GWG) and the birth weight and length of their offspring. We aimed to determine the association between DEB with GWG and the weight and length of adolescents’ offspring. We conducted a study with 379 participants. To evaluate DEB, we applied a validated scale. We identified three factors from DEB by factorial analysis: restrictive, compensatory, and binge–purge behaviors. The main events were GWG and offspring’s birth weight and length. We performed linear regression models. We found that 50% of adolescents have at least one DEB. Excessive and insufficient GWG were 37 and 34%, respectively. The median GWG was 13 kg; adolescents with restrictive behaviors had higher GWG (13 vs. 12 kg, *p* = 0.023). After adjusting for pregestational body mass index and other covariables, the restrictive (β = 0.67, *p* = 0.039), compensatory (β = 0.65, *p* = 0.044), and binge–purge behaviors (β = 0.54, *p* = 0.013) were associated with higher GWG. We did not find an association between the birth weight and length of newborns with DEB, and suggest that DEB is associated with GWG but not with the birth weight or length of the offspring.

## 1. Introduction

Adolescent pregnancy is a social and public health problem. Worldwide, 11% of births are among adolescents, and most of them come from low- or middle-income countries [1]. Pregnancy during adolescence implies higher risks for fetal and maternal health [2]; for instance, rates of low and excessive gestational weight gain (GWG) can reach up to 65% [3,4]. In addition, pregnant adolescents can be a vulnerable group because they have not finished their physical growth, and their neonates may present a higher risk of alterations in birth weight and length.

It is known that diet can have an important role in GWG. Therefore, to avoid adverse outcomes for pregnant women, their gestational weight gain should be monitored, with nutritional counselling during pregnancy and other factors, such as physical activity and lifestyle. Unfortunately, however, there are no data on adolescent pregnancy [5].

Regarding the evidence of the effects of pregestational body mass index (pBMI) and GWG on maternal and neonatal outcomes, it has been reported that being underweight before pregnancy increases the risk for preterm birth and for delivering a small for gestational age (SGA) newborn [6]. On the other hand, overweight and obesity are high-risk factors for gestational diabetes, hypertensive syndrome, and fetal growth disorders [7]. Concerning weight gain, women with insufficient gestational weight gain may experience anemia [8]. Conversely, those with excessive weight gain are at an elevated risk of cesarean delivery, preeclampsia, gestational diabetes, blood transfusions, weight retention after delivery, and long-term obesity [9]. Furthermore, excessive GWG has been associated with overweight and obesity in childhood and adolescence of the offspring [10].

Among Mexican adolescents, the prevalence of disordered eating behaviors (DEB) has increased in recent years [11], and in 2016, 50% had at least one DEB. Female adolescents have a higher probability of having these behaviors [12]. During pregnancy, some of the DEBs were less severe or occurred with less frequency [13]. However, this statement is not conclusive due to a lack of research in adolescent pregnancy. Evidence about GWG, birth weight and length, and eating disorders (ED) has focused on adult pregnant populations [14,15,16,17,18,19], whose ED prevalence has been between 0.1 and 5%. Having an ED during pregnancy can jeopardize maternal and neonatal health. For example, offspring of adolescent mothers have higher rates of low or large gestational age; while in mothers, there is an increased risk of miscarriage, preterm birth [17], preeclampsia [18], and inadequate GWG.

Few studies have reported the frequency of DEB during pregnancy [19,20]. In addition, information about the association of DEB with perinatal outcomes in adolescent pregnancy is scarce. Although DEB has less severity than ED, DEB frequency is high in adolescents [21]. Therefore, research in this area is essential.

DEB might affect the adolescents’ maternal weight and their neonates’ weight and length at birth. On the one hand, some restrictive eating behaviors can reduce the intake of energy and nutrients; therefore, lower growth can be expected. On the other hand, the same restrictive eating behaviors also activate neuroendocrine signals, stimulating hyperphagia [22] and resulting in higher birth weight and length, and maternal weight gain.

A limitation of studies that analyzed the association of DEB with maternal and neonatal outcomes [23,24,25] was that they did not adjust their analysis by pregestational weight. Pregestational weight can be a confounding factor because heavier women tend to have higher DEB frequency and weight gain [26]. Thus, in this study, we aimed to determine the association between the presence of different DEBs with gestational weight gain and the weight and length of Mexican adolescents’ offspring.

## 2. Materials and Methods

We carried out a prospective follow-up study (2014–2019) at the National Institute of Perinatology (Instituto Nacional de Perinatología, INPer) in Mexico, a tertiary care center in Mexico City. Most patients were women from low- and low–middle income households who lacked social security coverage and lived in neighboring states of Mexico City. The inclusion criteria were pregnant adolescents between 12 and 19 years old with a first and singleton pregnancy, and received antenatal care and delivery at INPer. Participants were excluded if they had any substance dependence; had autoimmune, infectious, or pregestational metabolic diseases; or were vegan. Moreover, if participants were diagnosed with any disease (e.g., gestational diabetes) during the follow up, they were eliminated from the study since they were receiving special medical and nutritional care that impacted our main study variables.

Regarding sample size, the present study is part of a bigger investigation project with the purpose of estimating the presence of disordered eating behaviors (DEBs) and eating habits in pregnant adolescents. For this reason, we included all the participants that were recruited for the original objective in our analysis.

### 2.1. Disordered Eating Behavior Evaluation

Trained personnel evaluated for DEB using a validated scale for the Mexican population [27] with acceptable internal consistency (Cronbach’s alpha 0.72–0.83). This scale consists of 10 Likert items that measure body image and restrictive and compensatory eating practices in the previous three months. The Likert items responses were scored from zero (never or rarely) to three (very frequently). A total score ≥10 was considered as the presence of DEB. We performed an exploratory factorial analysis to identify whether there were patterns regarding DEB (see Table 1). Three factors emerged: Factor 1 was called binge–purge (items 5, 6, 8, 9, and 10); Factor 2 was labeled as restrictive behaviors (items 1, 6 and 7); and Factor 3 included compensatory behaviors (items 2, 3, and 4). For each factor, we generated a score which was derived from the sum of answers to questions included in each factor (Table 1).

### 2.2. Anthropometric Evaluation

We performed all anthropometric measurements following Lohman’s techniques [28]. Self-reported pregestational weight was inquired by trained personnel. This weight corresponded to the weight of the participant at least three months before pregnancy. Height was measured with a manual stadiometer (SECA 222, Hamburg, Germany 0.1 cm accuracy) at the beginning of the study. With pregestational weight and height in the first antenatal consultation, we calculated pregestational body mass index (pBMI); then, pBMI was categorized according to the percentiles derived from the World Health Organization growth charts for BMI for sex and age. The percentile pBMI was classified as follows: <3, low weight; between ≥3 and <85, normal weight; between ≥85 and <97, overweight; and ≥97, percentile obesity [29].

We registered the last gestational weight one week before delivery using a digital scale (TANITA, Tokyo, Japan, model BWB-800; 0.010 kg accuracy). We calculated GWG by the difference between the last gestational weight and the pregestational weight. GWG was divided into three categories: insufficient, if the weight was below the recommendation; adequate, if the weight gain was within the recommendation; and excessive, if the weight gain was above the recommendation. 

Neonatal anthropometric measurements were obtained in the first 24 h after birth. Weight was obtained and registered using a digital pediatric scale (SECA 374, Hamburg, Germany. Model Baby and Mommy, 1 g accuracy). We obtained the length in cm using an infantometer, (SECA 416, Hamburg, Germany, 0.1 cm accuracy).

Other variables were obtained from clinical records, such as gestational age in weeks and type of delivery: cesarean section or vaginal delivery. We registered socio-demographic information to characterize our sample: chronological age; education level: elementary, middle school, and high school; marital status: single, married, and cohabitation. Finally, to determine the socioeconomic level, we used a questionnaire validated for the Mexican population; the resulting categories within our sample were middle, low–middle and low [30].

### 2.3. Ethical Aspects

This research was approved by the Institutional Ethics, Biosafety, and Research Committee (number 212250-49541-INPer). All adolescents and their guardians were informed of the study’s objectives and procedures involved, emphasizing the voluntary nature of their potential participation. We obtained written informed assent from the adolescents and their guardians or parents. A numeric code identifying each adolescent mother was used as a guarantee of confidential data collection and analysis.

### 2.4. Statistical Analysis

According to the distribution of the continuous variables, we calculated central tendency measurements to characterize our sample. Regarding categorical variables, we calculated relative and absolute frequencies. To compare frequencies of GWG and the weight and length of offspring at birth according to DEB factors, we performed the Mann–Whitney U test, Kruskal–Wallis test, or Student’s *t*-test. We also compared frequencies with Pearson’s Chi-square test. We calculated four linear regression models for DEB factors that could be associated with GWG and the birth weight and length of the offspring. We calculated a crude model, and three models were adjusted by potential confusing variables. Statistical significance was considered with a *p*-value < 0.050. All analyses were performed using software Stata/v.SE16.1 (College Station, TX, USA).

## 3. Results

A total of 379 participants were included in the study. The mean age was 15.9 years, and 19% were overweight/obese. The most common socioeconomic level was low–middle, and the majority of participants were single. All maternal and neonatal characteristics are shown in Table 2.

Nearly 50% of all adolescents reported at least one DEB; the most common DEB was restrictive behavior, followed by binge eating behavior. Half the participants were worried about becoming fat or eating too much; meanwhile, four out of ten presented control loss over what they ate and/or excessively exercised (Table 1).

**Figure 1:**
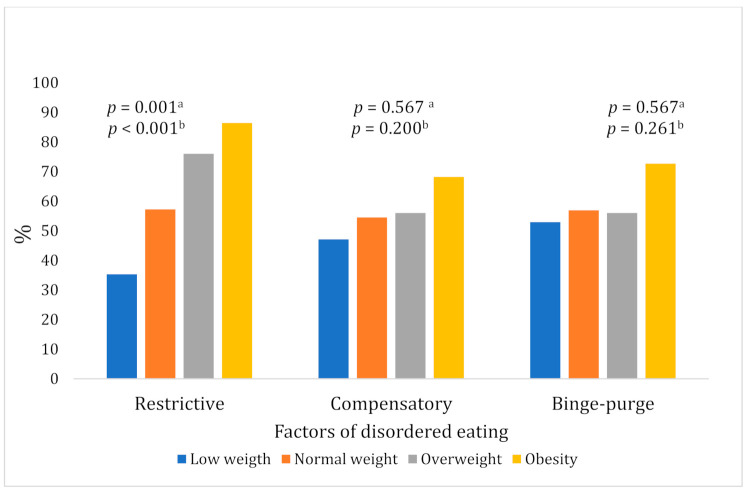
Disordered eating behaviors according to the pregestational BMI. ^a^ *p*-value based on Pearson’s Chi Square; ^b^ *p*-value based on linear association Chi-square test.

Compared with adolescents of normal weight, those with overweight or obesity had a greater frequency of the three types of DEBs during their pregnancy, but statistical significance was only observed for the comparison between pBMI and restrictive DEB (see Figure 1).

Thirty-seven percent of the participants had excessive GWG, and 34.3% had insufficient GWG. Excessive GWG was more frequent among adolescents with restrictive behaviors; GWG categories were no different in the compensatory and binge–purge behaviors. Regarding the weight and length of offspring at birth, no differences were observed between the pBMI categories nor the three types of DEBs (see Table 3). Nevertheless, it is important to note that the birth weight of offspring of adolescents with pregestational obesity tended to be lower than other pBMI categories (Table 3).

Finally, according to linear regression models, higher DEB scores in compensatory strategies were associated with GWG only in the adjusted Model 4; restrictive behavior score was associated with GWG in the crude and adjusted models; and binge–purge behaviors were associated with higher GWG only in the three adjusted models. In contrast, restrictive behaviors were positively associated with birth weight only in the adjusted models 2 and 3. None of the DEBs were associated with birth length (Table 4).

## 4. Discussion

In our study with pregnant adolescents, the most frequent DEB was restrictive behavior, followed by binge eating behavior. Excessive and insufficient GWGs were the most frequent. Adolescents with restrictive behaviors had higher GWG. The restrictive, compensatory, and binge–purge behaviors were associated with higher GWG.

The more frequent items were “I have worried about getting fat”, “Sometimes I have eaten too much, that I have been stuck on food” and “I’ve lost control of what I eat”. These results are similar to those obtained in a representative sample of Mexican non-pregnant adolescents [31] with frequencies of 40.2, 61.1, and 29.7, respectively [13]. In addition, the high frequency of DEB in non-pregnant adolescents might be associated with body image dissatisfaction, especially among those who are overweight [21].

Our findings suggest that DEB persisted during pregnancy in adolescents. In contrast, in adult women diagnosed with eating disorders, their eating disorders decreased during the first trimester of pregnancy [24]. Nevertheless, they experienced remissions of binges in the last trimester, especially among adult women with overweight or obesity [32]. The difference between the persistence of DEB in pregnant adolescents compared to the decrease in eating behaviors in pregnant adults may be due to those eating disorders being more severe and chronic conditions than DEB [33]. The persistence of DEB during adolescent pregnancy might be attributed to the fact that restrictive and compensatory behaviors have been normalized; therefore, the behaviors are perceived as less severe or not problematic.

In the present study, excessive and insufficient GWG were the most common categories, appearing in four and three out of ten adolescents, respectively, similar to other studies performed with young and low-income women [34,35,36,37]. In our sample of pregnant adolescents from Mexico City, less than 30% had adequate GWG, which also coincides with several reports from the United States and Canada [3,38,39]. These findings and trends should draw attention to the potential adverse effects of inadequate GWG on health in young women, suggesting the implementation of timely interventions to promote adequate GWG.

We observed a relationship between restrictive, compensatory, and binge–purge behaviors and higher GWG, even after adjusting for potential confounding variables, similar to findings among Norwegian women with ED [25,26]. These results may seem counterintuitive because restrictive behaviors and practices should lead to lower food intake and, therefore, lower weight gain. A probable explanation for our finding may be that the decontrol between restriction and subsequent binge–purge episodes carries forward to an excessive energy intake [23] and higher GWG.

Among adult women with ED, it is expected that their neonates have either low or high birth weight [19]. Women with ED have an energy intake so low or excessive, before or during pregnancy, that it may affect the birth weight and length of the offspring [15,16]. On the contrary, in our study, we did not observe an association between DEB and the birth weight and length of the offspring of pregnant adolescents. The lack of association could be because DEB severity is not enough to affect the maternal energy and nutrient storage.

According to the most recent Mexican National Survey on Health and Nutrition, female adolescents have a high intake of low-quality food and high sedentary level. In addition, more than 35% of women in this age group are overweight/obese. Therefore, adolescents who experience an early pregnancy with excessive gestational weight gain and with any DEB may be at risk of overweight and obesity in the future [40].

## 5. Limitations and Strengths

We must recognize that we used a convenient sample of a unique health center in terms of external validity. Therefore, the possibility of making any generalization is limited. At the same time, this hospital is a reference center at a regional level in Mexico. Therefore, the population that received antenatal care is heterogeneous in terms of geographical areas where women live. Hence, our results should not be generalized to other social and demographic contexts.

The design of our study has some qualities that improve internal validity, although there are some biases. We used a prospective longitudinal design, which allowed us to guarantee that the exposition preceded the outcomes. Most previous studies had cross-sectional design [25,39]. As in any observational research, we cannot exclude the possibility that a confounding variable explains the associations that we observed. Comparability of adolescent mothers with or without DEB was increased using multiple linear regression models adjusted for relevant confounders (e.g., pBMI, see below). With these models, we hope that some unmeasured characteristics were partially matched between adolescent mothers with or without DEB.

Another major limitation of our study was that we did not include neonatal and maternal adverse outcomes since they could be potential confounding factors for our principal variables.

On the other hand, because the sample size was not calculated within our main objective, we performed the power calculation for each linear regression model per outcome variable, that is, GWG, birth weight, and birth length, using the software G*Power v3.1 (Faul, Erdfelder, & Buchner, 2009, Düsseldorf, Germany). The input parameters for the two-tailed post-hoc tests were as follows: 0.05 precision; effect sizes of 0.54, 2.44, and 0.01; a total sample size of 363; and six predictors; the calculated power was 99%, 99%, and 49%, for GWG, birth weight, and birth length, respectively.

To our knowledge, our study is the first to explore and analyze the association of DEB with GWG in pregnant adolescents, and the birth weight and length of their offspring, taking into account the confusing role of pBMI. As previously reported [20,27,29], in our sample, women with DEB had higher pBMI. Pregnant adolescents who worried about becoming fat turned to restrictive strategies often, and they had higher pBMI. Therefore, models were required to be adjusted by pBMI to control its effect on the association of DEB and GWG.

The current research focuses on a scarcely explored age group, despite its physiological, emotional, and social vulnerability. Our study generated novel information to serve clinical and research personnel to continue this line of inquiry.

Our findings offer a first step to comprehensive antenatal care for adolescents in health facilities. It may be a call to action for health authorities, particularly considering the high prevalence of DEB and low frequency of adequate GWG. Many pregnant adolescents reported concern about gaining weight, mainly among those with a higher BMI, which could generate a vicious circle—the higher the BMI, the greater the risk for DEB practice, leading to greater body weight [25].

DEB should be identified, monitored, and controlled by the health sector through the provision of advice from qualified health personnel to adolescents, to try to achieve adequate GWG. Otherwise, adolescents will incur a risk of high retention of postpartum weight [41]. A reason for the above could be the lack of professional advice during pregnancy.

## 6. Conclusions

In a group of adolescent mothers, restrictive, compensatory, and binge–purge behaviors were associated with higher GWG. Additionally, pBMI was associated with DEB. However, DEB was not associated with the weight and length of the offspring at birth.

Our findings suggest that most adolescents keep maintain DEB during pregnancy, leading to excess weight problems due to the consequences of binge behavior, such as the accumulation of weight at an early age.

## Figures and Tables

**Table 1:** Items and factorial analysis of the disordered eating behaviors inventory in a sample of Mexican adolescent pregnant women (*n* = 379).

	Answers to DEBs Items						
	N-AN	ST	F	VO	Factors		
Items	%	%	%	%	F1	F2	F3
Eigen-value					2.93	1.94	1.65
% variance					29.2	19.4	16.5
1. I have worried about getting fat	45.4	41.4	7.9	5.3	−0.09	0.83	0.10
2. Sometimes I have eaten too much, that I have been stuck on food	51.7	37.7	8.2	2.4	0.09	−0.13	0.85
3. I’ve lost control of what I eat	75.2	16.4	5.8	2.6	−0.06	0.15	0.78
4. I have vomited after eating, to try to lose weight	97.6	1.6	0.5	0.3	−0.09	0.16	0.45
5. I have fasted to try to lose weight	95.2	3.3	1.3	0.2	0.53	0.29	0.17
6. I have been dieting to try lose weight	91.8	6.9	0.8	0.5	0.54	0.42	−0.11
7. I have exercised to try to lose weight	73.1	22.1	2.9	1.8	0.14	0.72	−0.11
8. I have used pills to try to lose weight	96.6	2.1	0.8	0.5	0.79	0.05	0.02
9. I have used diuretics to try to lose weight	97.9	1.8	0.0	0.3	0.83	−0.06	−0.00
10. I have taken laxatives to try to lose weight	98.6	1.1	0.0	0.3	0.85	−0.09	0.01

N-AN: never or almost never. ST: sometimes. F: frequently. VO: very often. F1: binge–purge behaviors. F2: restrictive behaviors. F3: compensatory behaviors.

**Table 2:** Characteristics of pregnant adolescents and their offspring (*n* = 379).

Variables	Mean ± SD	Minimum-Maximum
Maternal		
Age (years)	15.9 ± 1.3	12–19
Menarche age (years)	11.5 ± 1.3	7–16
Pregestational weight (kg) ^a^	50 (46–59)	28–100
Height (cm)	156 ± 5.6	139.4–176
Pregestational BMI ^a^	21.2 (19–23)	13.5–39.1
Percentile BMI WHO ^a^	57.8 (31–75)	0–100
pBMI	Low weight ^b^	17 (4.5)
	Normal weight	290 (76.5)
	Overweight	50 (13.2)
	Obesity	22 (5.8)
Socioeconomic level ^b^	Middle	44 (11.6)
	Low-middle	220 (58.0)
	Low	115 (30.3)
Marital status ^b^	Single	217 (57.3)
	Married	18 (4.8)
	Living together	144 (37.7)
Education level ^b^	Elementary	96 (25.5)
	Middle school	238 (63.1)
	High school	43 (11.4)
**Perinatal characteristics**		
Gestational age (weeks) ^a^	39 (38–40)	26.6–41.3
Gestational weight gain (kg) ^a^	13 (8.3–17)	−7.75–35.5
Delivery ^b^	Cesarean section	162 (43.1)
	Vaginal	217 (56.9)
**Neonate**		
Birth weight (g)	2950 (2690–3235)	1030–4105
Length (cm) ^a^	50 (48–51)	31–56
Gender ^b^	Girl	172 (45.4)
	Boy	207 (54.6)

^a^ Data are presented as median (percentile 25–percentile 75); ^b^ data are presented as frequency (%). SD: standard deviation. BMI: body mass index. WHO: World Health Organization. pBMI: pregestational body mass index.

**Table 3:** Maternal gestational weight gain and neonatal birth weight and length according to maternal pregestational BMI and factors of disordered eating behaviors.

			Gestational Weight Gain (%) Percent						Neonatal Measurements at Birth			
Variables	Categories	*n*	GWG in kg ^a^	*p*-Value	Insufficient GWG ^b^	Adequate GWG ^b^	Excessive GWG ^b^	*p*-Value	Birth Weight in g ^a^	*p*-Value	Birth Length in cm ^a^	*p*-Value
Pregestational BMI	Underweight	17	14.2 (13.8–19)	0.003 ^c^	11.8	58.8	29.4	<0.001 ^d^	2889 (2605–3155)	0.071 ^c^	50 (47.5–51)	0.101 ^c^
	Normal weight	290	13.0 (9.0–17.0)		41.7	29.7	28.6		2915 (2670–3225)		49 (48–51)	
	Overweight	50	12.7 (7.9–18.6)		24.0	20.0	56.0		3027 (2825–3340)		50 (49–51)	
	Obesity	22	10.0 (4.4–13.0)		27.3	9.1	63.6		2107 (2900–3335)		50 (49–51)	
Restrictive behaviors	Present	229	13.5 (9–17.9)	0.003 ^e^	33.2	25.8	41.0	0.003 ^d^	2978 (2700–3250)	0.237 ^e^	50 (48–51)	0.927 ^e^
	Absent	150	12.0 (8–15.5)		43.3	32.7	24.0		2902 (2685–3208)		50 (48–51)	
Compensatory behaviors	Present	209	13.0 (9.0–17)	0.451 ^e^	34.4	29.7	35.9	0.469 ^d^	2925 (2685–3250)	0.692 ^e^	49 (48–50)	0.777 ^e^
	Absent	170	12.5 (8.0–17)		40.6	27.1	31.4		2955 (2710–3225)		50 (48–51)	
Binge-purge behaviors	Present	218	13.0 (8.8–17)	0.559 ^e^	34.9	30.3	34.9	0.503 ^d^	2915 (2676–3250)	0.447 ^e^	49 (48–50)	0.754 ^e^
	Absent	161	12.5 (8.2–17)		40.5	26.1	33.5		2958 (2720–3225)		50 (48–51)	

^a^ Data are presented as median (percentile 25–percentile 75); ^b^ data are presented as percentage; ^c^ *p*-value based on Kruskal–Wallis test; ^d^ *p*-value based on Pearson’s Chi-square test; ^e^ *p*-value based on Mann–Whitney U test. GWG: gestational weight gain. BMI: body mass index.

**Table 4:** Linear regression models of the association of gestational weight gain, birth weight and length, with disordered eating behaviors.

Variables	M1		M2		M3		M4	
	B	*p*-Value	B	*p*-Value	B	*p*-Value	B	*p*-Value
**Gestational weight gain**								
Compensatory behaviors	0.34	0.265	0.38	0.241	0.42	0.194	0.65	0.044
Restrictive behaviors	0.57	0.016	0.58	0.021	0.54	0.032	0.67	0.039
Binge-purge behaviors	0.41	0.077	0.65	0.014	0.60	0.023	0.65	0.013
Total	0.35	0.008	0.40	0.004	0.38	0.006	0.54	0.000
**Birth weight**								
Compensatory behaviors	−10.80	0.643	14.28	0.456	16.86	0.387	32.95	0.582
Restrictive behaviors	19.22	0.279	30.29	0.036	30.74	0.036	24.58	0.110
Binge-purge behaviors	−10.45	0.550	−18.20	0.201	−18.16	0.253	−20.21	0.203
Total	2.94	0.769	5.77	0.481	6.49	0.432	2.44	0.774
**Birth length**								
Compensatory behaviors	−0.04	0.776	0.10	0.380	0.13	0.270	0.12	0.317
Restrictive behaviors	−0.00	0.981	0.06	0.454	0.06	0.495	0.06	0.516
Binge-purge behaviors	−0.06	0.789	−0.06	0.467	−0.08	0.390	−0.08	0.392
Total	−0.01	0.908	0.02	0.813	0.01	0.796	0.01	0.845

M1: crude model. M2: adjusted model by maternal age and gestational age. M3: adjusted model by maternal age, gestational age, and socioeconomic status. M4: Adjusted model by maternal age, gestational age, and maternal pBMI. B: beta coefficient.

## Data Availability

The data presented in this study are available from the corresponding author upon reasonable request.

## Abbreviations

GWG	Gestational weight gain
BMI	Body mass index
DEB	Disordered eating behaviors
INPer	National Institute of Perinatology
pBMI	Pregestational body mass index
ED	Eating disorder

## References

[1] United Nations Fund for Population Activities Girlhood, Not Motherhood (2015). Preventing Adolescent Pregnancy. https://www.unfpa.org/sites/default/files/pub-pdf/Girlhood_not_motherhood_final_web.pdf.

[2] De Azevedo W.F., Diniz M.B., da Fonseca E.S.V.B., de Azevedo L.M.R., Evangelista C.B. (2015). Complications in Adolescent Pregnancy: Systematic Review of the Literature. Einstein.

[3] Whelan E., Armson B.A., Ashley-Martin J., MacSween K., Woolcott C. (2017). Gestational Weight Gain and Interpregnancy Weight Change in Adolescent Mothers. J. Pediatr. Adolesc. Gynecol..

[4] Campos C.A.S., Malta M.B., Neves P.A.R., Lourenço B.H., Castro M.C., Cardoso M.A. (2019). Gestational Weight Gain, Nutritional Status and Blood Pressure in Pregnant Women. Rev. Saude. Publica..

[5] Tsakiridis I., Kasapidou E., Dagklis T., Leonida I., Leonida C., Bakaloudi D.R., Chourdakis M. (2020). Nutrition in Pregnancy: A Comparative Review of Major Guidelines. Obstet. Gynecol. Surv..

[6] Ratnasiri A.W.G., Lee H.C., Lakshminrusimha S., Parry S.S., Arief V.N., DeLacy I.H., Yang J.-S., Dilibero R.J., Logan J., Basford K.E. (2019). Trends in maternal prepregnancy body mass index (BMI) and its association with birth and maternal outcomes in California, 2007–2016: A retrospective cohort study. PLoS ONE.

[7] Voerman E., Santos S., Inskip H., Amiano P., Barros H., Charles M.-A., Chatzi L., Chrousos G.P., Corpeleijn E., LifeCycle Project-Maternal Obesity and Childhood Outcomes Study Group (2019). Association of Gestational Weight Gain With Adverse Maternal and Infant Outcomes. JAMA.

[8] Figueiredo A.C.M.G., Gomes-Filho I., Batista J.E.T., Orrico G.S., Porto E.C.L., Pimenta R.M.C., Conceição S.D.S., Brito S.M., Ramos M.D.S.X., Sena M.C.F. (2019). Maternal anemia and birth weight: A prospective cohort study. PLoS ONE.

[9] Sun Y., Shen Z., Zhan Y., Wang Y., Ma S., Zhang S., Liu J., Wu S., Feng Y., Chen Y. (2020). Effects of pre-pregnancy body mass index and gestational weight gain on maternal and infant complications. BMC Pregnancy Childbirth.

[10] Elwan D., Olveda R., Medrano R., Wojcicki J.M. (2021). Excess pregnancy weight gain in latinas: Impact on infant’s adiposity and growth hormones at birth. Prev. Med. Rep..

[11] Schaumberg K., Welch E., Breithaupt L., Hübel C., Baker J.H., Munn-Chernoff M.A., Yilmaz Z., Ehrlich S., Mustelin L., Ghaderi A. (2017). The Science behind the Academy for Eating Disorders’ Nine Truths About Eating Disorders. Eur. Eat. Disord. Rev..

[12] Villalobos A., Unikel C., Hernández-Serrato M.I., Bojórquez I. (2020). Disordered eating in Mexican adolescents, 2006–2018. Salud. Publica. Mex..

[13] Harrison M.E., Balasubramanaiam B., Robinson A., Norris M.L. (2018). Adolescent Pregnancy and Eating Disorders: A Minireview and Case Report. Eat. Weight Disord..

[14] Easter A., Bye A., Taborelli E., Corfield F., Schmidt U., Treasure J., Micali N. (2013). Recognising the Symptoms: How Common Are Eating Disorders in Pregnancy?. Eur. Eat. Disord. Rev..

[15] Dörsam A.F., Preißl H., Micali N., Lörcher S.B., Zipfel S., Giel K.E. (2019). The Impact of Maternal Eating Disorders on Dietary Intake and Eating Patterns during Pregnancy: A Systematic Review. Nutrients.

[16] Micali N., Northstone K., Emmett P., Naumann U., Treasure J.L. (2012). Nutritional Intake and Dietary Patterns in Pregnancy: A Longitudinal Study of Women with Lifetime Eating Disorders. Br. J. Nutr..

[17] Micali N., Stemann Larsen P., Strandberg-Larsen K., Nybo Andersen A.-M. (2016). Size at Birth and Preterm Birth in Women with Lifetime Eating Disorders: A Prospective Population-Based Study. BJOG.

[18] Watson H.J., Zerwas S., Torgersen L., Gustavson K., Diemer E.W., Knudsen G.P., Reichborn-Kjennerud T., Bulik C.M. (2017). Maternal Eating Disorders and Perinatal Outcomes: A Three-Generation Study in the Norwegian Mother and Child Cohort Study. J. Abnorm. Psychol..

[19] Oliboni C.M., Alvarenga M.D.S. (2015). Eating attitudes, attitudes related to weight gain, and body satisfaction of pregnant adolescents. Rev. Bras. Ginecol. Obstet..

[20] Sonneville K.R., Calzo J.P., Horton N.J., Haines J., Austin S.B., Field A.E. (2012). Body Satisfaction, Weight Gain and Binge Eating among Overweight Adolescent Girls. Int. J. Obes..

[21] Zullig K.J., Matthews-Ewald M.R., Valois R.F. (2016). Weight Perceptions, Disordered Eating Behaviors, and Emotional Self-Efficacy among High School Adolescents. Eat. Behav..

[22] Dulloo A.G., Jacquet J., Montani J.-P., Schutz Y. (2015). How Dieting Makes the Lean Fatter: From a Perspective of Body Composition Autoregulation through Adipostats and Proteinstats Awaiting Discovery. Obes. Rev..

[23] Fairburn C.G., Stein A., Jones R. (1992). Eating Habits and Eating Disorders during Pregnancy. Psychosom. Med..

[24] Zerwas S.C., Von Holle A., Perrin E.M., Cockrell Skinner A., Reba-Harrelson L., Hamer R.M., Stoltenberg C., Torgersen L., Reichborn-Kjennerud T., Bulik C.M. (2014). Gestational and Postpartum Weight Change Patterns in Mothers with Eating Disorders. Eur. Eat. Disord. Rev..

[25] Bulik C.M., Von Holle A., Siega-Riz A.M., Torgersen L., Lie K.K., Hamer R.M., Berg C.K., Sullivan P., Reichborn-Kjennerud T. (2009). Birth Outcomes in Women with Eating Disorders in the Norwegian Mother and Child Cohort Study (MoBa). Int. J. Eat. Disord..

[26] Allen K.L., Byrne S.M., McLean N.J., Davis E.A. (2008). Overconcern with weight and shape is not the same as body dissatisfaction: Evidence from a prospective study of pre-adolescent boys and girls. Body Image.

[27] Unikel-Santoncini C., Bojórquez-Chapela I., Carreño-García S. (2004). Validation of a brief questionnaire to measure the risk of abnormal eating behaviors. Salud. Publica. Mex..

[28] Pelletiet D. (1992). Anthropometric Standardization Reference Manual: Abridged Edition. Edited by T.G. Lohman, A.F. Roche, and R. Martorell. vi 90 Pp. Champaign, IL: Human Kinetics Books. 1991. U.S. 15.00, Canada 18.50 (paper). Am. J. Hum. Biol..

[29] WHO (2006). Child Growth Standards: Length/Height-for-Age, Weight-for-Age, Weight-for-Length, Weight-for-Height and Body Mass Index-for-Age: Methods and Development.

[30] Socioeconomic Level Index of the Mexican Association of Market Research and Public Opinion Agencies (AMAI) September 2014 AMAI Regulation NSE 8 × 7. www.amai.org/NSE/NivelSocioeconomicoAMAI.pdf.

[31] Barriguete-Meléndez J.A., Unikel-Santoncini C., Aguilar-Salinas C., Córdoba-Villalobos J.A., Shamah T., Barquera S., Rivera J.A., Hernández-Avila M. (2009). Prevalence of Abnormal Eating Behaviors in Adolescents in Mexico: Mexican National Health and Nutrition Survey 2006. Salud. Publica. Mex..

[32] Kolko R.P., Emery R.L., Marcus M.D., Levine M.D. (2017). Loss of Control over Eating before and during Early Pregnancy among Community Women with Overweight and Obesity. Int. J. Eat. Disord..

[33] Mackenna M.J., Escaffi M.J., González T., Leiva M.J., Cruzat C. (2021). Trastornos de La Conducta Alimentaria En El Embarazo. Rev. Méd. Clín. Las Condes..

[34] Gould Rothberg B.E., Magriples U., Kershaw T.S., Rising S.S., Ickovics J.R. (2011). Gestational Weight Gain and Subsequent Postpartum Weight Loss among Young, Low-Income, Ethnic Minority Women. Am. J. Obstet. Gynecol..

[35] Chang T., Moniz M.H., Plegue M.A., Sen A., Davis M.M., Villamor E., Richardson C.R. (2017). Characteristics of Women Age 15–24 at Risk for Excess Weight Gain during Pregnancy. PLoS ONE.

[36] Ekambaram M., Irigoyen M., DeFreitas J., Rajbhandari S., Geaney J.L., Braitman L.E. (2018). Gestational Weight Gain among Minority Adolescents Predicts Term Birth Weight. World J. Pediatr..

[37] Groth S.W., Holland M.L., Smith J.A., Meng Y., Kitzman H. (2017). Effect of Gestational Weight Gain and Prepregnancy Body Mass Index in Adolescent Mothers on Weight and Body Mass Index of Adolescent Offspring. J. Adolesc Health.

[38] Elchert J., Beaudrot M., DeFranco E. (2015). Gestational Weight Gain in Adolescent Compared with Adult Pregnancies: An Age-Specific Body Mass Index Approach. J. Pediatr..

[39] Gonçalves S., Freitas F., Freitas-Rosa M.A., Machado B.C. (2015). Dysfunctional Eating Behaviour, Psychological Well-Being and Adaptation to Pregnancy: A Study with Women in the Third Trimester of Pregnancy. J. Health Psychol..

[40] Shamah-Levy T., Vielma-Orozco E., Heredia-Hernández O., Romero-Martínez M., Mojica-Cuevas J., Cuevas-Nasu L., Santaella-Castell J.A., Rivera-Dommarco J. (2020). Encuesta Nacional de Salud y Nutrición 2018–19: Resultados Nacionales.

[41] Saarikko J., Niela-Vilén H., Rahmani A.M., Axelin A. (2021). Identifying Target Behaviors for Weight Management Interventions for Women Who Are Overweight during Pregnancy and the Postpartum Period: A Qualitative Study Informed by the Behaviour Change Wheel. BMC Pregnancy Childbirth.

